# Use of RT-PCR in conjunction with a respiratory pathogen assay to concurrently determine the prevalence of bacteria and SARS-CoV-2 from the nasopharynx of outpatients

**DOI:** 10.3389/fepid.2023.1274800

**Published:** 2023-11-01

**Authors:** James F. Shurko, Robert B. Page, Chris A. Mares, Vivian Nguyen, Kristina Lopez, Niti Vanee, Pramod K. Mishra

**Affiliations:** ^1^ Molecular Diagnostics Division, iGenomeDx, San Antonio, TX, United States; ^2^ Department of Life Sciences, Texas A&M University-San Antonio, San Antonio, TX, United States; ^3^ Lousiana Scholars’ College, Northwestern State University, Natchitoches, LA, United States

**Keywords:** COVID-19, co-detection, bacteria, nasopharynx, outpatient

## Abstract

**Introduction:**

COVID-19 has emerged as a highly contagious and debilitating disease caused by the SARS-CoV-2 virus and has claimed the lives of over 7.7 million people worldwide. Bacterial co-infections are one of many co-morbidities that have been suggested to impact the outcome of COVID-19 in patients. The goals of this study are to elucidate the presence of bacteria in the nasopharynx of SARS-CoV-2 positive and negative patients and to describe demographic categories that may be associated with the detection of these organisms during one of the initial waves of the COVID-19 pandemic.

**Methods:**

To this end, we investigated SARS-CoV-2 and bacterial co-detection from outpatient RT-PCR testing in Texas.

**Results:**

The results indicate that *Staphylococcus aureus*, *Streptococcus pneumoniae*, *Klebsiella pneumoniae*, *Moraxella catarrhalis*, and *Haemophilus influenzae* were the most frequently detected bacteria in both SARS-CoV-2 positive and SARS-CoV-2 negative patients and that these bacteria were present in these two patient populations at similar proportions. We also detected *Staphylococcus aureus* in a significantly larger proportion of males relative to females and people under 65 years of age relative to those 65 and over. Finally, we observed that SARS-CoV-2 was more commonly detected in Hispanics compared to non-Hispanics; however, low disclosure rates make volunteer bias a concern when interpreting the effects of demographic variables.

**Discussion:**

This study describes the bacteria present in the nasopharynx of SARS-CoV-2 positive and negative patients, highlights associations between patient demographics and SARS-CoV-2 as well as bacterial co-detection. In addition, this study highlights RT-PCR based molecular testing as a tool to detect bacteria simultaneously when SARS-CoV-2 tests are performed.

## Introduction

Coronavirus disease 2019 (COVID-19) is caused by Severe Acute Respiratory Syndrome Coronavirus 2 (SARS-CoV-2) and represents an important global health challenge. Approximately 7.7 million deaths have been reported as due to COVID-19 globally with over 1.1 million occurring in the United States (1, 2). In the state of Texas alone, the CDC has reported a total of over 8.5 million cases and over 93 thousand deaths since March of 2020. As of April 5th, 2023, the CDC reports an incidence of over 1.7 thousand cases per day contributing to an average of twelve daily deaths (3). While vaccines are available to the general public in the United States, nearly 30.6% of the general population is not fully vaccinated (defined as receiving two doses of either the Pfizer or Moderna vaccine or a single dose of the Johnson and Johnson vaccine) and in the state of Texas, approximately 29% of the population is not fully vaccinated (4, 5). These data, combined with the discovery of newly emerging strains, demonstrate that COVID-19 is a continuing health concern (6).

SARS-CoV-2 infections display a wide range of prognoses ranging from asymptomatic infection to severe respiratory failure and death. More specifically, patients may experience fever, cough, fatigue, dyspnea, diarrhea, loss of taste or smell, aches, conjunctivitis, pneumonia, acute respiratory distress syndrome (ARDS) and multi-organ failure (7, 8). Treatments for COVID-19 were initially limited but multiple vaccine candidates and antiviral or immunomodulatory drugs have been tested since the beginning of the pandemic (9–12). In addition to comorbidities such as hypertension and diabetes, bacterial co-infections have been shown to have a profound impact on the outcomes of patients infected with SARS-CoV-2 (13–16). Subsequently, empiric antimicrobial therapy has been commonly administered to affected patients. Widespread antibiotic use remains controversial however, due to increased healthcare costs, adverse drug reactions, and the evolution of antimicrobial resistance. This study therefore aims to identify associations between patient demographics (age, sex, race and ethnicity) and the frequency of SARS-CoV-2 and bacterial co-detection as well as compare the prevalence of common respiratory bacteria present within the nasopharynx of SARS-CoV-2 negative and SARS-CoV-2 positive patients. By elucidating the frequency and species of bacteria, as well as the demographics most commonly associated with co-detection, this study may add insight that can serve as a reference for reviews or surveillance studies, provide a stepping stone for comprehensive clinical studies, and demonstrate the ability of reverse transcription-polymerase chain reaction (RT-PCR) based molecular tests to detect organisms concurrently with SARS-CoV-2 testing.

## Methods

### Study design, sample collection & patient population

This study was a retrospective, multicenter analysis of patient samples obtained from April 2020 to April 2021. Each patient sample consisted of a single specimen collected from the nasopharynx of individuals. A single nasopharyngeal swab was used to collect each specimen and only one specimen was collected from each patient. Both SARS-CoV-2 and respiratory bacteria testing were conducted from the same patient sample. Patient samples included in this study were collected from a total of 57 outpatient clinics, doctor's offices, and nursing homes within the state of Texas. While patient samples were not collected from hospitals, no other restrictions were placed on the facilities from which they were collected. Inclusion criteria consisted of patients of any sex or age who experienced symptoms consistent with SARS-CoV-2 infection or other respiratory infections. Example indications consisted of unspecified upper respiratory infection, cough, fever, bronchiolitis, wheezing, throat pain and fatigue. Requisition forms containing ICD10 codes and face sheets were reviewed to determine patient eligibility. Since the study aimed to identify differences in bacteria detected in SARS-CoV-2 positive and negative patients at a given time point, only patients receiving both SARS-CoV-2 and respiratory bacteria testing concurrently using a single nasopharyngeal swab for both tests were included. Asymptomatic patients who received tests solely due to contact with an infected individual or those who received repeat tests for monitoring purposes were also excluded. Finally, patients that underwent either respiratory or SARS-CoV-2 testing alone were excluded. Given the retrospective nature of the study and the fact that only symptomatic patients were indicated for respiratory testing, healthy controls were not assessed.

Patient sample collection kits were composed of requisition forms and E-swabs (Copan Diagnostics, Murrieta, CA) or nasopharyngeal swabs (Huachenyang iClean, Shenzhen, China) with conical tubes (Stellar Scientific, Baltimore Maryland) containing storage buffer and were sent to outpatient facilities. Storage buffer was prepared according to CDC guidelines and all components including the conical tubes were DNAase and RNAse free to preserve the integrity of the organisms and nucleic acids (17). Trained clinicians including nurses and medical assistants obtained patient samples from the nasopharynx of each person. Additionally, patient demographics including sex, date of birth, ethnicity and race were self-reported by patients and included on requisition forms. Prior to analysis, patient information was de-identified using the Safe Harbor Method (18). The study was conducted using secondary de-identified human data. This study was exempt and therefore consent was waived as approved by the Institutional Review Board at Texas A&M University-San Antonio (TAMUSA IRB #2022-016). All methods were carried out in accordance with all relevant guidelines and regulations.

### RNA isolation and bacteria identification

Bacteria and SARS-CoV-2 were detected using RT-PCR. Primers, probes, and enzymes used to detect SARS-CoV-2 were purchased from Integrated DNA Technologies (Coralville, Iowa). The Fast Track Diagnostics (FTD) respiratory pathogen 33 assay (Selma, Malta) was used to detect bacteria and is currently distributed by Siemens Healthineers as catalog# 11373926 (Erlangen, Germany). After nasopharyngeal swabs were obtained, RNA was extracted using the Applied Biosystems MagMax Viral/Pathogen II kit (ThermoFisher, Waltham, MA) as per the manufacturer's instructions. After isolation, RNA was amplified and detected via RT-PCR using the Applied Biosystems QuantStudio 7 flex and 12K flex systems (ThermoFisher, Waltham, MA). Positive SARS-CoV-2 tests were defined as a cycle threshold (Ct) value of 40 or less for RNAse P, N1 and N2 collectively in line with the FDA's emergency use authorization (19, 20). The FTD respiratory pathogen 33 assay contained probes for *Bordetella spp, Chlamydia pneumoniae, Haemophilus influenzae, Haemophilus influenzae type B, Klebsiella pneumoniae, Legionella pneumophila, Moraxella catarrhalis, Mycoplasma pneumoniae, Salmonella spp, Staphylococcus aureus, and Streptococcus pneumoniae*. Bacterial detection was defined as positive when RT-PCR tests resulted in Ct values <35 for any of the respective probes.

### Statistical analysis

**Figure 1:**
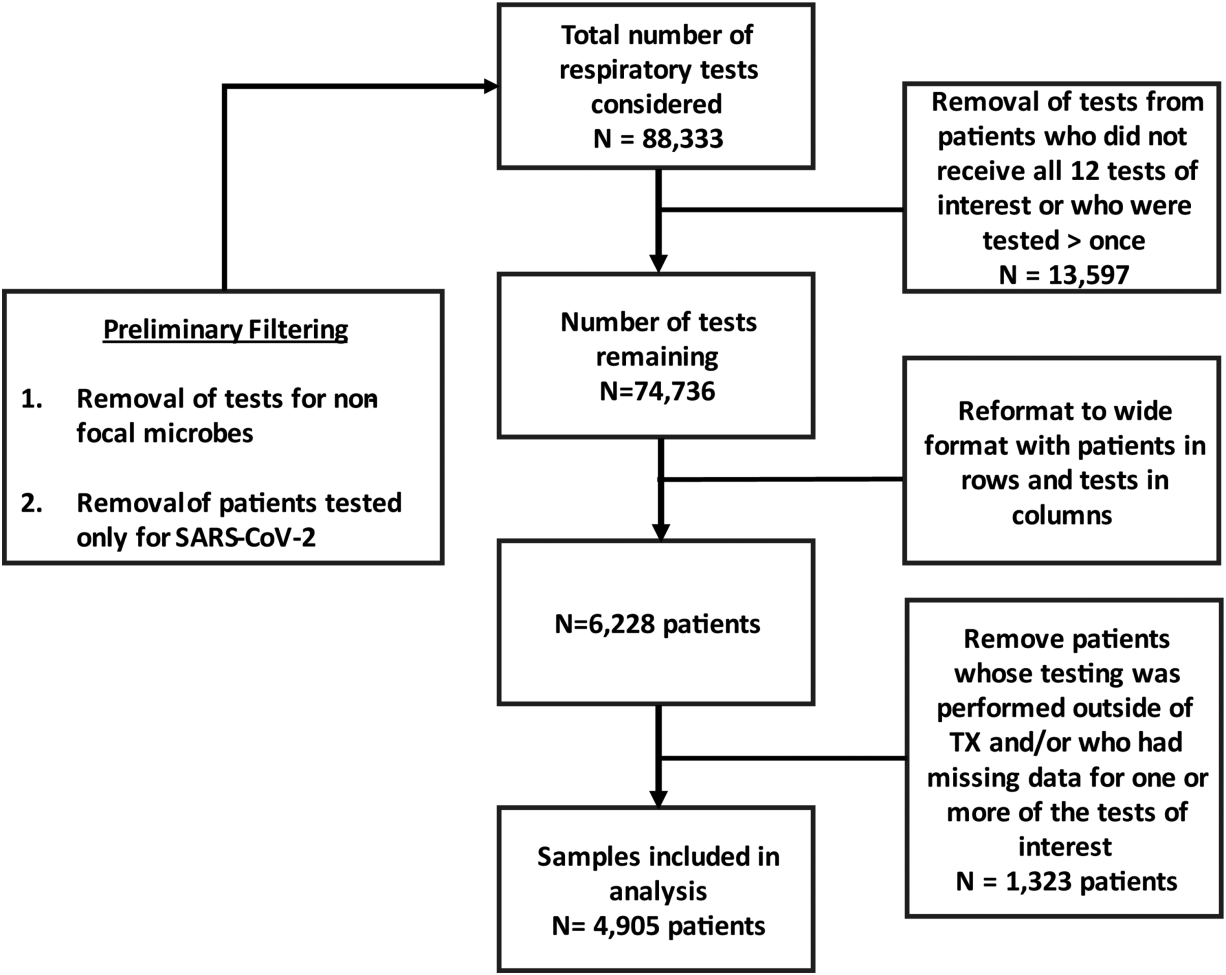
Flow chart depicting data formatting based on inclusion and exclusion criteria. Tests refer to individual targets such as a single virus, bacterium or fungus.

The data set analyzed in this study comprised of patient samples submitted for both SARS-CoV-2 and respiratory bacteria testing. Prior to analysis, individual tests (defined as a single virus or bacterium) and patients were filtered based on the scheme described in Figure 1. Briefly, samples subjected only to SARS-CoV-2 testing were removed, as were tests corresponding to viruses and/or bacteria that were not among the 12 microorganisms examined in this study. Following this filtering step, 88,333 individual tests spread across 7,037 patients remained. To ensure that analyses were based on a common suite of tests, only patients tested for SARS-CoV-2 and all 11 respiratory bacteria described above were included. Additionally, to ensure that all observations were independent, patients that were continually monitored or otherwise tested on multiple occasions were excluded resulting in a total of 74,736 individual tests spread across 6,228 patients. Given the differences in mitigation policy between states, and the fact that Texas was the most heavily represented state in this data-set, we focused on patients residing in Texas for this study. After imposing these filters, the analyses we conducted were based on 4,905 patients in total. While these data are composed of patients from throughout the state of Texas, 1,522 (31%) of the patients sampled were sampled in the McAllen-Edinburgh-Mission metropolitan statistical area (MSA) and 2,357 (48%) patients were sampled in the San Antonio-New Braunfels MSA. Thus, 79% of the patients that our analyses are based on come from these two MSAs. In addition, these data include 1,026 patients who did not disclose their city of residence, were from MSAs (e.g., Dallas-Fort Worth) other than McAllen-Edinburgh-Mission and San Antonio-New Braunfels, or rural areas throughout the state.

We used the “binom.test” function in R to calculate point estimates and 95% confidence intervals for the proportion of patients positive for SARS-CoV-2, *S. aureus*, and bacteria other than *S. aureus*. This approach was applied across several demographic categories and was complemented by using R to test for equal proportions (“prop.test” function) among: (1) races, (2) ethnicities, (3) women vs. men, and (4) patients ≥ 65 vs. patients <65. We then adjusted the 12 resulting *P*-values (three virus/bacteria categories x four demographic factors) for multiple testing using the False Discovery Rate (FDR = 0.05) correction described by Benjamini and Hochberg (21).

In addition to examining positivity rates across demographic factors, we also used “binom.test” to compute point estimates and 95% confidence intervals of the proportion of SARS-CoV-2 positive and SARS-CoV-2 negative patients who tested positive for each of the eight bacterial species detected in our survey. We then used “prop.test” to make comparisons between the bacterial positivity rates of SARS-CoV-2-positive and SARS-CoV-2-negative patients for bacterial species that had sufficient data to ensure that the assumptions associated with expected cell counts were met. The resulting five *P*-values were adjusted for multiple testing by controlling the FDR at the 0.05 level (21).

Finally, we used the odds ratio (*OR*) to estimate the magnitude and direction of association between: (1) SARS-CoV-2 and detection of bacterial species of any kind, (2) SARS-CoV-2 and detection of bacteria other than *S. aureus*, (3) SARS-CoV-2 and detection of more than one bacterium of any kind, and (4) SARS-CoV-2 and detection of more than one kind of bacteria, not including *S. aureus*. The null hypothesis that *OR* = 1.00 was assessed via Fisher's Exact Test and 95% confidence intervals of *OR* were computed using the “fisher.test” function in R. The four resulting *p-*values were corrected for multiple testing by controlling the FDR at the 0.05 level (21).

## Results

A demographic summary of the overall dataset and the patients that were SARS-CoV-2 negative and positive respectively is presented in Table 1. Table 2 shows point estimates and 95% confidence intervals for the proportion of patients from various demographics (i.e., sex, age, ethnicity, and race) that were positive for SARS-CoV-2, *S. aureus*, and bacteria other than *S. aureus*. Of the 12 tests conducted to compare positivity across these demographics, three were statistically significant after correcting for multiple testing (Supplementary Material File S1). For example, the positivity rate for SARS-CoV-2 was 10.8 percentage points (25.3% vs. 14.5%) higher in Hispanics compared to non-Hispanics (Table 2); however, over half of the patients in our study did not disclose their ethnicity (Table 1), so this result should be interpreted carefully. As another example, the positivity rate for *S. aureus* was 5.3 percentage points (28.6% vs. 23.3%) higher in males than females and 5.1 percentage points (26.3% vs. 21.2%) higher in patients under 65 years of age when compared to individuals 65 years of age or older (Table 2). Finally, there were no statistically significant differences in the positivity rates of non-*S. aureus* bacteria detected across any of the demographic variables examined.

**Table 1:** Number and percentage of individuals in the study in various demographic categories and the number and percentage of SARS-CoV-2 positive and SARS-CoV-2 negative patients that belong to each respective category.

Demographic	Total (%)	SARS-CoV-2 negative	SARS-CoV-2 positive
Sex			
**Female**	2,837 (57.8)	2,287 (58.3)	550 (55.8)
Male	2,014 (41.1)	1,590 (40.6)	424 (43.1)
Undisclosed sex	54 (1.1)	43 (1.1)	11 (1.1)
Age			
≥65 years of age	846 (17.2)	663 (16.9)	183 (18.6)
<65 years of age	4,037 (82.3)	3,237 (82.58)	800 (81.2)
Undisclosed age	22 (0.45)	20 (0.51)	2 (0.2)
Race			
African American	65 (1.33)	53 (1.4)	12 (1.2)
Asian	36 (0.73)	31 (0.79)	5 (0.51)
Native American	7 (0.14)	6 (0.15)	1 (0.10)
Pacific Islander	1 (0.02)	1 (0.03)	0 (0.0)
White	1,892 (38.6)	1,442 (36.8)	450 (45.7)
Undisclosed race	2,904 (59.2)	2,387 (60.9)	517 (52.5)
Ethnicity			
Hispanic	1,983 (40.4)	1,481 (37.8)	502 (51.0)
Non-Hispanic	283 (05.8)	242 (6.2)	41 (4.2)
Undisclosed ethnicity	2,639 (53.8)	2,197 (56.1)	442 (44.9)
Total (denominator)	4,905 (100)	3,920 (100)	985 (100)

**Table 2:** Percentage of patients who tested positive for SARS-CoV-2 or respiratory pathogens stratified by demographic.

Demographic	Number of patients	Percentage (95% Confidence interval)		
	*n*	SARS-CoV-2	*S. aureus*	Other^a^
Sex				
Female	2,837	19.4 (18.0–20.9)	**23.3** (**21.8–24.9)**	9.2 (8.1–10.3)
Male	2,014	21.1 19.3–22.9)	**28.6** (**26.6–30.6)**	10.3 (9.0–11.7)
Undisclosed sex	54	20.4 (10.6–33.5)	20.4 (10.6–33.5)	5.6 (1.2–15.4)
Age				
≥65 years of age	846	21.6 (18.9–24.6)	**21.2** (**18.5–24.1)**	7.8 (6.1–9.8)
<5 years of age	4,037	19.82 (18.6–21.1)	**26.3** (**25.0–27.7)**	9.9 (9.0–10.9)
Undisclosed age	22	9.1 (1.1–29.2)	27.3 (10.7–5.0)	22.7 (7.82–45.4)
Race				
African American	65	18.5 (9.9–30.0)	27.7 (17.3–40.2)	10.8 (4.4–20.9)
Asian	36	13.9 (4.7–29.5)	30.6 (16.4–48.1)	5.6 (0.7–18.7)
Native American	7	14.3 (0.36–57.9)	28.6 (3.7–71.0)	28.6 (3.7–70.96)
Pacific Islander	1	0.0 (0.0–97.5)	0.0 (0.0–97.5)	0.0 (0.0–97.5)
White	1,892	23.8 (21.9–25.8)	27.8 (25.7–29.8)	9.6 (8.2–10.9)
Undisclosed race	2,904	17.8 (16.4–19.4)	23.8 (22.3–25.4)	9.7 (8.6–10.8)
Ethnicity				
Hispanic	1,983	**25.3** (**23.4–27.3)**	25.5 (23.6–27.5)	8.7 (7.5–10.1)
Non-Hispanic	283	**14.5** (**10.6–19.1)**	31.5 (26.1–37.2)	10.6 (7.3–14.8)
Undisclosed ethnicity	2,639	**16.8** (**15.3–18.2)**	24.7 (23.1–26.4)	10.2 (9.0–11.4)
**Total**	4,905			

Percentages and 95% confidence intervals (95% CI) of patients with SARS-CoV-2, *S. aureus* and

^a^ Other bacteria contained in the respiratory pathogen assay regardless of *S. aureus* presence. Statistically significant findings are bolded.

**Figure 2:**
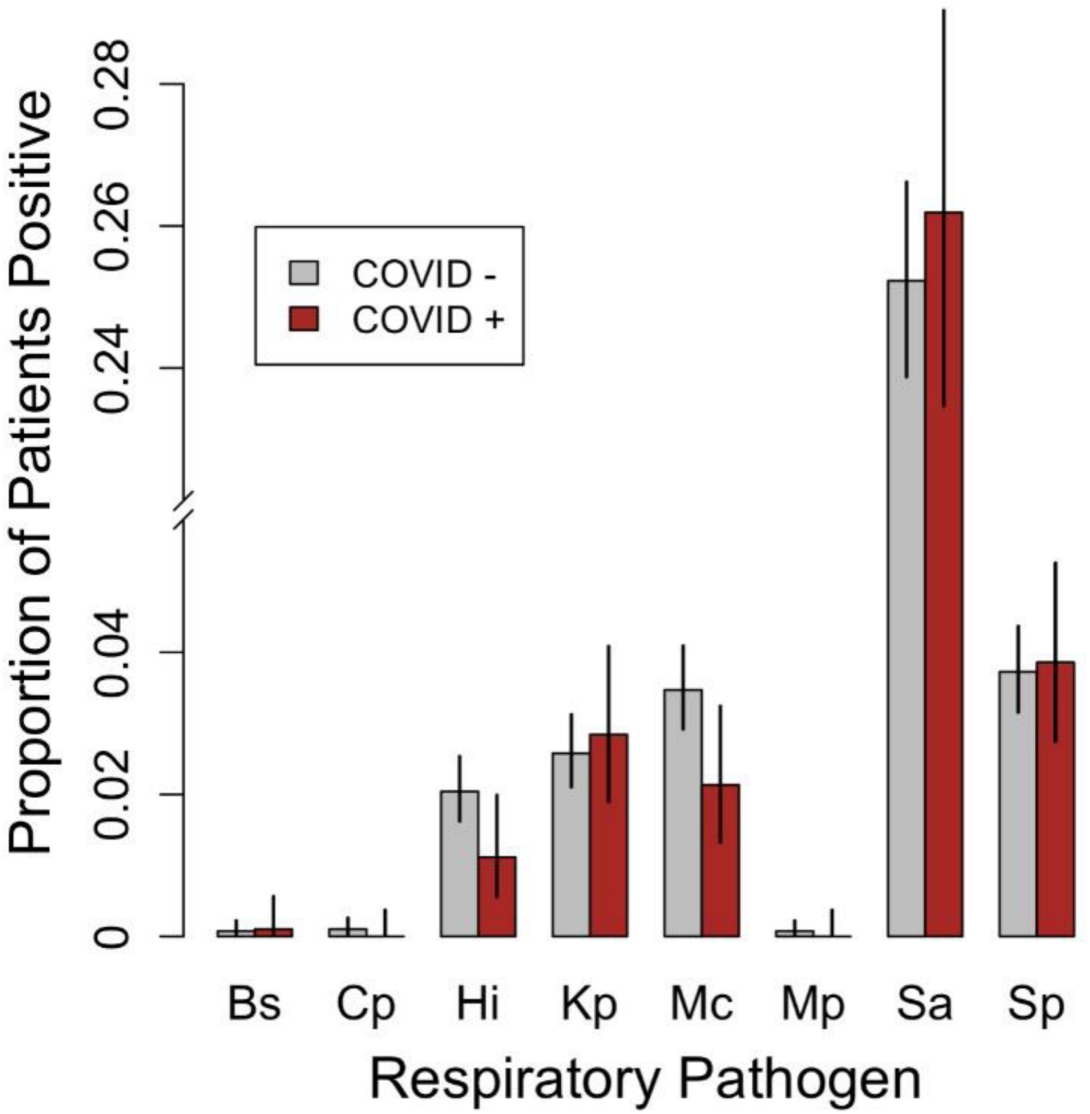
Grouped bar plot comparing the proportions of SARS-CoV-2 negative patients (COVID −; *n* = 3,920) and SARS-CoV-2 [positive patients (COVID +; *n* = 985) that tested positive for the various respiratory bacterial species detected during the study period. Error bars represent 95% confidence intervals for the estimated proportions. Bs, *Bordetella sp*., Cp, *C. pneumoniae*, Hi, *H. influenzae*, Kp, *K. pneumoniae*, Mc, *M. catarrhalis*, Mp, *M. pneumoniae*, Sa, *S. aureus*, Sp, *S. pneumoniae*. Note that *H. influenzae B*, *L. pneumophila*, *and Salmonella spp*. were not detected in any patients and were therefore omitted.

A total of eight bacterial species were detected in this study including *S. aureus* (*n = *1,247)*, S. pneumoniae* (*n = *184)*, M. catarrhalis* (*n = *157)*, K. pneumoniae* (*n = *129)*, H. influenzae* (*n = *91)*, M. pneumoniae* (*n = *3)*, C. pneumoniae* (*n = *4) *and Bordetella sp* (*n = *4)*.* The estimated proportions and 95% confidence intervals of SARS-CoV-2 positive and SARS-CoV-2 negative patients who tested positive for each of these eight bacterial species are shown in Figure 2. Three of these eight bacterial species (*Bordetella* sp., *C. pneumoniae*, and *M. pneumoniae*) were detected in fewer than five patients; therefore, tests of equal proportions were not performed. Of the remaining five species, tests comparing positivity rates in SARS-CoV-2 negative and SARS-CoV-2 positive patients were not statistically significant after correcting for multiple testing (Supplementary Material File S1).

Of the 4,905 patients included in this study 1,566 (31.9%) tested positive for at least one bacterium and 471 (9.6%) patients tested positive for a bacterium other than *S. aureus.* Additionally, 213 (4.3%) patients tested positive for multiple bacteria and 85 (1.7%) patients tested positive for multiple bacteria not counting S. *aureus* (Table 3). Contingency tables, odds ratios, 95% confidence intervals, and *P*-values from Fisher's Exact Tests assessing the association between SARS-CoV-2 and bacterial detection of any kind, bacterial detection other than *S. aureus*, polymicrobial detection including *S. aureus*, and polymicrobial detection not including *S. aureus* are shown in Table 3. Upon correcting for multiple testing, no significant associations were identified. However, the percentage of patients in which multiple non-*S.aureus* bacteria were detected was more than twice as high in SARS-CoV-2 negative patients (1.9%) when compared to SARS-CoV-2 positive (0.91%) patients (Table 3).

**Table 3:** Association between SARS-CoV-2 status and number of patients that tested positive for the various bacterial respiratory pathogens detected during the study period.

** **	COVID status		OR (95% CI)	*p*-value	Adjusted *p*-value
One or more bacteria of any kind	Positive	Negative	** **		** **
Yes	309	1,257			
No	676	2,663	** **		
Percentage	31	32	0.9684 (0.8302–1.1281)	0.7023	0.9306
One or more bacteria excluding *S. aureus*					
Yes	87	384	** **		** **
No	898	3,536	** **		
Percentage	8.8	9.8	0.8921 (0.6907–1.1426)	0.3970	0.7940
Multiple bacteria of any kind					
Yes	42	171			
No	943	3,749			
Percentage	4.3	4.4	0.9765 (0.6743–1.3871)	0.9306	0.9306
Multiple bacteria excluding *S. aureus*					
Yes	9	76			
No	976	3,844			
Percentage	0.91	1.9	0.4665 (0.2047–0.9374)	0.02806	0.11224

Raw counts, odds ratios (OR), 95% confidence intervals (95% CI), and *p*-values based on Fisher's exact test of the null hypothesis that the true odds ratio is equal to one. Odds ratios describe how many times more likely bacterial infection is given the presence of SARS-CoV-2.

## Discussion

Among patients infected with SARS-CoV-2 globally, meta-analyses report a wide range (1%–100%) of antimicrobial prescribing rates. In the United States these rates generally range from 50% to 95% and are typically near 70% (22–25). While some of these patients do suffer from bacterial co-infection or develop secondary infections after hospital admission, a disparity may exist between the rate of prescribing antibiotics and the number of patients concurrently infected with SARS-CoV-2 and bacteria. As antibiotics are prescribed, adverse drug reactions and antimicrobial resistance become concerns. With studies identifying drug resistant organisms in SARS-CoV-2 patients treated with antibiotics, it has become imperative to determine the necessity of utilizing these agents (26, 27). This study therefore aimed to identify differences in bacteria present among patients with and without SARS-CoV-2.

The rate of co-detection varies widely in the literature ranging from approximately 1% to 50% (23, 26, 28–34). Many factors may contribute to this variation including differences in geography, setting (inpatient vs. outpatient), time of collection (upon admission vs. during hospital stay), site of collection/sample type (sputum vs. bronchiolar lavage vs. nasopharyngeal swab etc.), and targeted organisms (bacteria vs. viruses or both). This study detected bacteria in 31.9% of patients including 31% among SARS-CoV-2 positive patients and 32% among SARS-CoV-2 negative patients. While this bacterial co-detection rate may be higher than those estimated from many studies, it is typical within the literature for studies utilizing nasopharyngeal swabs to observe higher rates of bacteria compared to those drawing from other sources such as sputum. For example, a study conducted by Calcogno et al., in which 75.9% of samples were collected via nasopharyngeal swabs, reported a similar rate (32.7% of patients overall in comparison to 31.9% in our study) (31). Additionally, Calcogno et al. identified *S. aureus* in 15.4% of patients while *S. aureus* was identified in 25.4%% of patients in our study. Several sources indicate that *S. aureus* is observed in the nares of approximately 20%–30% of the population (35–37). Therefore, it is likely that the rate of detection was driven largely by commensal bacteria.

In addition to *S. aureus* our study identified *H. influenza* (1.8%), *K. pneumoniae* (2.6%), *S. pneumoniae* (3.8%) and *M. catarrhalis* (3.2%) as the most common species. Similarly, in a meta-analysis by Musuuza et al., the most common species detected in patients co-infected with SARS-CoV-2 were *K. pneumoniae* (9.9%), *S. pneumoniae* (8.2%), *S. aureus* (7.7%), *H. influenzae* (6.6%), and *M. pneumoniae* (4.3%), and *M. catarrhalis* (1.7%) (38). Unlike in our study however, Musuuza et al. found that *M. pneumoniae* comprised 4.3% of the bacterial species identified, whereas our study only detected three instances of *M. pneumoniae*, all of which were SARS-CoV-2 negative patients. While these organisms were detected less frequently than *S. aureus* it is worth noting that *Moraxella, Klebsiella* and *Haemophilus* have also been detected in the nares or nasopharynx of healthy participants (39, 40). Therefore, the possibility of commensals driving the prevalence of these organisms must also be considered when interpreting the results.

While many factors potentially contribute to the similarities and differences between our results and those of others, it is important to note that the types of bacteria observed may change over time and/or differ considerably between outpatient and hospital settings. For example, *Acinetobacter, Pseudomonas*, and *Enterococcus* were more commonly identified after prolonged hospital stays and multi-drug resistant organisms (MDROs) have been detected in hospitalized patients (26, 38). A limitation of comparing our results to the results of others is that the assay, and therefore the species targeted, often differs among studies. Thus, while it is expected for species associated with prolonged hospital stays to be less frequent in our outpatient population, use of the FTD respiratory pathogen 33 assay limited our ability to fully assess their impact because it does not target many of these organisms. Further, since the number of bacterial species detected was limited by this assay, the overall detection rate may have been limited as well. The overall detection rate may also be limited by symptomatic patients that did not see a doctor. This limitation was partially addressed by noting the presence of individual species. Additionally, separate analyses were conducted with and without *S. aureus.* In doing so, detection rates of specific bacteria could be compared across groups or between studies. Incorporating additional methodologies in future studies such as next generation sequencing (NGS) could further mitigate these limitations by revealing differences in the prevalence of organisms not included in the FTD respiratory pathogen 33 assay (41).

This study centered around SARS-CoV-2 and bacteria. The inclusion of fungi and other viruses would provide greater insight and possibly account for some instances where patients exhibit symptoms despite the absence of SARS-CoV-2 or bacteria. Despite these limitations, this study is unique in that few studies have specifically reviewed outpatient data and while several studies described the rate of co-infection in COVID-19 patients, few studied the difference in rate among SARS-CoV-2 positive and negative patients. Overall, this study, along with comparisons of our data with data from other studies, show little, if any evidence of an increased rate of bacterial co-detection among SARS-CoV-2 positive patients. While co-detection was similar among both groups, studies incorporating additional collection sites, culture and sensitivity, patient follow-up and outcome data would be invaluable to the delineation of co-infection and co-detection before guiding clinical decisions.

In addition to comparing the prevalence and types of bacteria detected in SARS-CoV-2 positive and negative patients, we sought to identify populations that may be associated with either SARS-CoV-2 or bacterial co-detection. In this data set, Hispanics were more commonly infected with SARS-CoV-2 when compared to non-Hispanics or when compared to those that did not self-report their ethnicity. This study was limited by these self-reported demographics however, and many patients declined to answer this question. Notably 2,639 patients did not disclose their ethnicity and only 283 patients identified as non-Hispanic compared to 1,983 patients who identified as Hispanic. Consequently, the ethnicity of 53.8% of the patients is unknown and therefore volunteer bias should be considered when interpreting these results. Despite this limitation, the proportion of Hispanics testing positive for SARS-CoV-2 is in line with data reported by the CDC (42). Others have reported similar observations, suggesting minorities have higher incidence and mortality rates due to COVID-19 (43–45). This observation is also supported by a recent review by Mackey et al. that points out that Hispanic patients suffered from higher rates of COVID-19 infection, hospitalization due to COVID-19 infection, and COVID-19 related mortality (46, 47). Further, age and sex were associated with *S. aureus* detection as it was more commonly identified in males and patients under the age of 65. The association of these demographics and *S. aureus* detection is in line with previous studies and is summarized in a review by Sollid et al. (48). No other associations between demographics and bacteria were identified in this study.

While immunological and sequence-based methods are valuable in detecting SARS-CoV-2, RT-PCR is currently the gold standard in the clinical setting as it is more sensitive than immunological-based methods and does not rely on the bioinformatic pipelines associated with NGS (41). As another consideration, step down therapy in the treatment of bacterial infections relies on the use of culture and sensitivity. Since this technique requires the isolation and growth of bacteria, results may take several days to receive. Using RT-PCR however, samples can be tested directly and results can be generated on the same day as processing. The dataset analyzed in this study was derived from patient samples which were concurrently tested for SARS-CoV-2 as well as bacteria from a single nasopharyngeal swab administered to a given patient. These data suggest that RT-PCR based molecular testing has the potential to identify bacteria while culture and sensitivities are being performed, though parallel studies comparing RT-PCR to culture and sensitivity are necessary to determine the extent to which these methodologies are congruent.

In summary, RT-PCR was used to concurrently test for bacteria and SARS-CoV-2 to ensure that no systemic discrepancies existed in nasopharyngeal sample collection and testing procedures. Using this approach, we found a higher rate of detection of SARS-CoV-2 in Hispanics; however, no significant differences were detected in the rate of bacterial co-detection between SARS-CoV-2 positive and SARS-CoV-2 negative patients. Despite the similarities in bacteria detected between these two groups, additional studies should be conducted prior to guiding therapy. We also detected *S. aureus* at higher rates in men and people under 65. However, *S. aureus* is commonly detected in the nares, suggesting that the presence of *S. aureus* in and of itself is not necessarily indicative of infection—an issue we addressed by conducting separate analyses that included and excluded *S. aureus.* Because this study was derived from outpatient settings at multiple sites within the state of Texas, these data may not generalize to populations from other parts of the US. However, by surveying outpatients, this study may be more applicable to the portion of the general public that does not require hospitalization. Moreover, since molecular tests are not contingent upon bacterial culture, they can be performed rapidly and may provide useful information prior to receiving results from culture and sensitivity.

## Data Availability

The data that support the findings of this study are available from the corresponding authors upon reasonable request.

## Supplementary material

SUPPLEMENTARY MATERIAL FILE S1

Excel workbook containing sheets showing (1) the results of tests assessing whether positivity rates for SARS-CoV2, *S. aureus*, and bacterial pathogens other than *S. aureus* differ among demographic factors (race, ethnicity, sex, and age) and (2) whether the positivity rates for individual bacterial pathogens differ among SARS-CoV2 positive and SARS-CoV2 negative patients.
